# Comparison of Outcomes of Internal Fixation Using Double-Angle Blade Plate Versus Double-Angle Dynamic Hip Screw With Pauwels Osteotomy in Neglected Fracture of the Neck of Femur

**DOI:** 10.7759/cureus.82677

**Published:** 2025-04-21

**Authors:** Spandan Mishra, Shivam Chawla, Sunit Pani, Tapan K Das

**Affiliations:** 1 Orthopaedics and Traumatology, Institute of Medical Sciences (IMS) and Siksha 'O' Anusandhan University Medical (SUM) Hospital, Bhubaneswar, IND; 2 Orthopaedics, Institute of Medical Sciences (IMS) and Siksha 'O' Anusandhan University Medical (SUM) Hospital, Bhubaneswar, IND

**Keywords:** double-angle blade plate, double-angle dhs, femur fracture, internal fixation, non-union, pauwels osteotomy

## Abstract

Introduction

Fracture of the neck of the femur is a significant orthopaedic injury with high morbidity and mortality rates, particularly among the elderly following low-velocity trauma. The complications of osteonecrosis and non-union are common in cases treated with any procedure of osteosynthesis. Surgical intervention through internal fixation is a common approach. Numerous procedures have been described for salvaging the femoral head to avoid or delay hip replacement in patients younger than 60 years. Operative methods to enhance vascularity and achieve healing at the fracture site include fixation with bone grafting, pedicle grafts to provide blood supply, or valgus intertrochanteric osteotomy. This paper compares the outcomes of two internal fixation techniques, double-angle blade plate (DABP) and double-angle dynamic hip screw (DHS) with Pauwels osteotomy, in the treatment of fracture neck of femur. We assess the outcomes based on functional recovery, complication rates, and overall patient satisfaction.

Materials and methods

Cases were selected from patients attending the OPD and Trauma Unit of IMS and SUM Hospital, Bhubaneshwar, from 2022 to 2023. The patients were divided into two groups: one group underwent internal fixation with a DABP, and the other group underwent fixation with a double-angle DHS with Pauwels osteotomy. All patients with a diagnosed fracture neck of femur who underwent internal fixation with either a DABP or double-angle DHS with Pauwels osteotomy, aged 17-60 years, and with a delay in presentation of more than three weeks, were included in the study. Patients aged over 60 years, with fresh fractures, pathological fractures, multiple fractures, or prior surgery on the affected hip, were excluded.

Results

A total of 30 patients were included in the study, with 16 in the DABP group and 14 in the double-angle DHS group. Both groups demonstrated improvement in Harris Hip Score (HHS) at 6-month and 12-month follow-ups, but the double-angle DHS group showed a slightly higher HHS, indicating faster functional recovery. The angle blade plate showed better stability and less bone removal. It allowed room for fibular grafting and early mobilization due to reliable fixation. However, it was technically more difficult to perform and was a less forgiving implant than DHS, which is technically simpler and provides compression at the fracture site. Healing time was similar in both groups. Overall, the results were markedly satisfactory, with good functional outcomes, active painless hip range of motion, and painless weight-bearing, with acceptable shortening in these cases managed with either double-angle DHS or DABP, each with some shortcomings.

Conclusion

This study concluded that Pauwels osteotomy and fixation with a double-angle DHS provided the patient with a relatively higher success rate concerning union, with acceptable limb length discrepancy and better functional outcomes in young adults with neglected fracture neck of femur. It serves as a head-salvaging procedure in properly selected patients.

## Introduction

Fractures of the neck of the femur are among the most serious injuries affecting the elderly population [1], often resulting from falls or underlying bone fragility. Given their complex anatomy and the high risk of complications such as avascular necrosis and non-union, these fractures present significant challenges to orthopaedic surgeons. Internal fixation is a standard treatment approach, with various techniques offering different advantages. The choice of fixation method can influence the outcome, recovery time, and overall patient satisfaction. Anatomical reduction and internal fixation as early as possible is typically recommended for young, active patients. When reduction and internal fixation were performed within six hours of the injury, Manninger J et al. [2] found a decreased incidence of femoral head collapse. However, due to poverty, illiteracy, and reliance on traditional bone setters, delayed presentation continues to be a significant issue in developing nations, where patients often report symptoms weeks after the fracture. Non-union is still observed in one-third of femoral neck fracture cases with displacement, even with advancements in surgical techniques and internal fixation devices. In such situations, several head-retaining techniques are used, including valgus osteotomy, internal fixation with vascularised grafts or free fibular-based grafting, and fixation using muscle pedicle bone grafting [3]. Valgus intertrochanteric osteotomy [4,5] alters the biomechanics at the fracture site. This study aims to compare two such techniques: double-angle blade plate (DABP) and double-angle dynamic hip screw (DHS) with Pauwels osteotomy. By examining the functional recovery, complication rates, and patient satisfaction associated with each method, we seek to provide valuable insights into the most effective treatment strategies for this debilitating injury.

## Materials and methods

**Figure 1:**
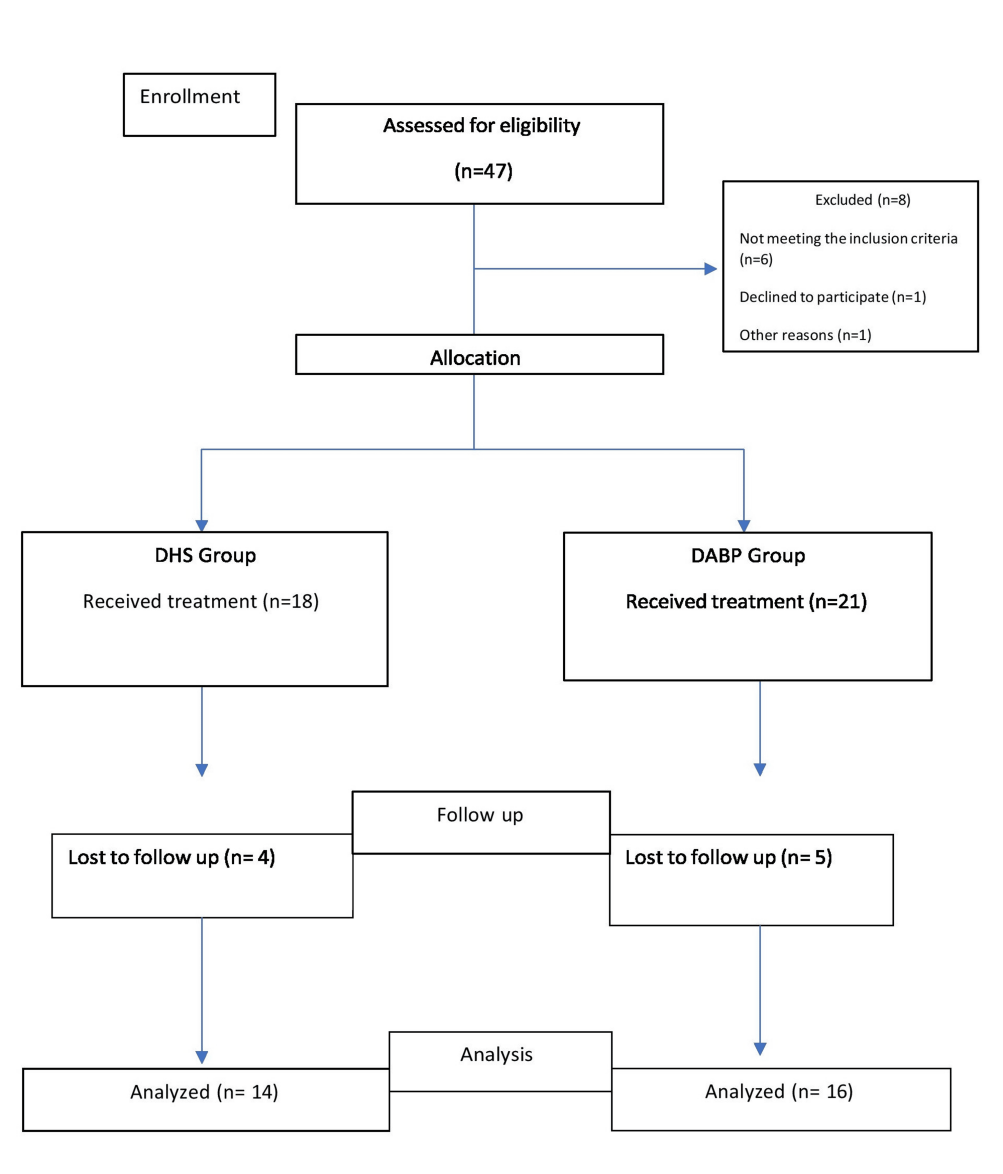
CONSORT flow diagram. DHS: Dynamic Hip Screw; DABP: Double-Angle Blade Plate; CONSORT: Consolidated Standards of Reporting Trials.

The study is a non-randomized interventional comparative study of patients who underwent internal fixation for fracture neck of femur at IMS and SUM Hospital from 2022 to 2023. The patients were divided into two groups: one group underwent fixation using a double-angle blade plate (DABP), and the other group underwent fixation using a double-angle DHS with Pauwels osteotomy. All patients with a diagnosed fracture neck of femur who underwent internal fixation with either a DABP or double-angle DHS with Pauwels osteotomy, aged 17-60 years, and with a delay in presentation of more than 3 weeks, were included in the study. Patients aged over 60 years, with fresh fractures, pathological fractures, multiple fractures, or prior surgery on the affected hip were excluded. The provided image (Figure 1) is a CONSORT flow diagram illustrating the progression of participants through different stages of the study comparing the DABP and the DHS treatment methods. Initially, 47 individuals were assessed for eligibility, out of which eight were excluded, six did not meet the inclusion criteria, one declined to participate, and another was excluded for other reasons. The remaining 39 participants were allocated into two groups: 18 individuals received treatment with the DHS, while 21 participants received treatment with the DABP. During the follow-up phase, four participants from the DHS group and five from the DABP group were lost to follow-up. Ultimately, 14 participants from the DHS group and 16 from the DABP group were included in the final analysis. This diagram provides a structured representation of participant flow, ensuring transparency in reporting and helping to identify potential biases in the study.

Pre-operative planning

Calculation of Wedge Angle

The Pauwels angle, also referred to as the shear angle, is the angle formed between the fracture plane and the horizontal plane, represented by a line contacting the roofs of the acetabulum. Pauwels identified an optimal shear angle ranging from 20° to 25° [6]. This calculation stems from Pauwels’ principle, which asserts that during any given instance, the weight subtended by the body projects onto the femoral head at an angle of 160 degrees. As a result, the compressive force is exerted perpendicular to this projected vector, translating to an angle of 20 degrees relative to the vertical (180° - 160° = 20°). The osteotomy or wedge angle is intended to achieve a final fracture plane angle of less than 30 degrees. Based on Pauwels’ principles, a fracture that forms an angle of 30 degrees or less will experience compressive forces, promoting union. Consequently, the angle of correction, or wedge angle, was calculated as follows:

Wedge angle (osteotomy angle) = Shear angle - Post-op desired angle (30 degrees)

Calculation of Pin Insertion Angle

The pin insertion angle is the angle formed between the edge of the pin situated in the femoral neck (initially located at the center of the femoral head at the beginning of the procedure) and the lateral cortex of the femur. Subsequently, the hip screw is positioned toward the center of the femoral head according to this predetermined pin insertion angle.

Pin insertion angle = Implant angle - Osteotomy angle

In all cases, surgery was performed on a fracture table under fluoroscopic guidance, with the patient in a supine position. The site of the fracture remained unexposed throughout the procedure. Each patient underwent an intertrochanteric osteotomy as outlined by Pauwels, which was achieved by performing an osteotomy at the upper level of the lesser trochanter, excising a lateral wedge of bone, and achieving valgus and lateral displacement of the distal fragment. Internal fixation was secured using either a 120° DABP or a double-angle DHS.

Surgical Technique for DABP Fixation

In our investigation, a 120° double-angled condylar blade plate was used to execute valgus osteotomy on 16 young patients (under 60 years of age) who had non-union of femoral neck fractures. A group of orthopaedic surgeons, under the direction of the first author, performed the surgery. To categorize the fractures using Pauwels' criteria, preoperative radiographs were taken. Patients with advanced avascular necrosis (AVN) on radiographs were excluded, and no preoperative MRI scans were performed.

Each fracture’s Pauwels angle was determined, and the osteotomy was designed to produce a Pauwels angle of 30° or less. Achieving compression at the fracture site was the primary goal. As explained by Pauwels F [6] and later modified by Mueller ME, the osteotomy was carried out at the lesser trochanter level, and the necessary wedge of bone was excised depending on the Pauwels angle [7].

Both spinal and epidural anesthesia were used during the procedure. The patient was positioned using an image intensifier guide on a standard fracture table. The greater trochanter was centered in a direct lateral cutaneous incision that extended to the femoral shaft. The proximal femur was exposed by splitting the vastus lateralis muscle and incising the fascia parallel to the skin incision. To temporarily stabilize the fracture, two 2.5 mm K-wires were inserted into the femoral head through the lateral cortex. To avoid compromising the blood supply to the femoral head, the fracture site was not opened during surgical exposure.

**Figure 2:**
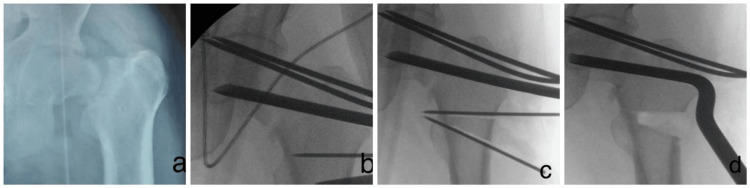
Introduction of chisel, marking of osteotomy site, and the osteotomy procedure. (a) Old neglected fracture of the neck of femur; (b) Marking of the osteotomy site; (c, d) Osteotomy.

**Figure 3:**
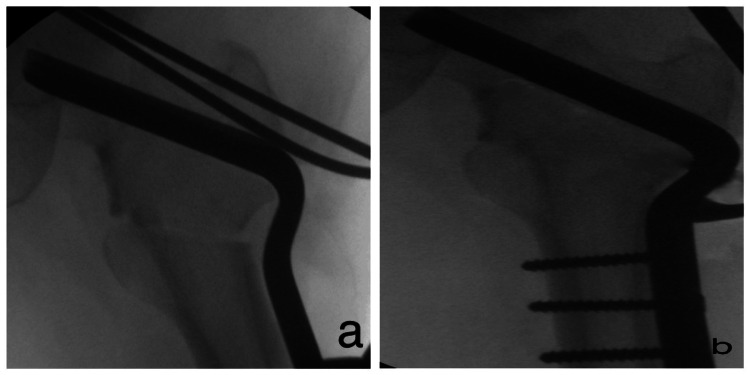
Plate insertion following limb abduction and wedge closure.

Using the image intensifier, the correct entry point of the chisel was marked on the greater trochanter in both anteroposterior and lateral views. A chisel was inserted into the femoral head to create a pathway for the blade of the 120° double-angled blade plate (Stainless Steel 316L, Medplus). The intertrochanteric osteotomy was performed 2 cm below the blade entry point, and the intended bone wedge was excised (Figure 2). The limb was abducted to close the osteotomy site, and the implant was aligned and secured with cortical screws (Figure 3).

After the surgery, patients were encouraged to use crutches and advised to refrain from putting weight on the operated leg for the initial six weeks. Weight-bearing was allowed after six weeks, depending on the patient’s comfort level. Complete weight-bearing was permitted only after verifying union. Clinical indications of a united fracture were characterized by the absence of pain or tenderness at the fracture site during weight-bearing, whereas radiological criteria included the presence of callus at the fracture site on standard radiographs.

Surgical Technique for Double-Angle DHS With Pauwels Osteotomy

Under anesthesia, the patient was placed supine on an orthopedic fracture table. A direct lateral approach was used to facilitate open reduction of the femoral fracture. Fluoroscopy was employed to guide the insertion of a guide pin for a DHS, positioned at a predetermined angle approximately 2 cm above the intended osteotomy site, targeting the center or infero-posterior quadrant of the femoral head. Following this, an appropriately sized DHS was inserted (Figure 3). At this juncture, the side plate was positioned such that it deviated from the femoral shaft, creating an angle that aligned with the planned osteotomy.

**Figure 4:**
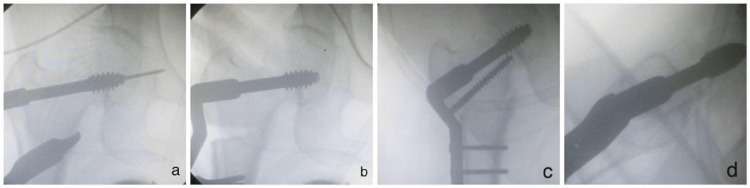
Insertion of guide wire, appropriate-sized DHS, osteotomy, and implant placement. (a) Insertion of guide wire and appropriate-sized screw; (b) Osteotomy; (c, d) Anteroposterior and lateral views after implant insertion. DHS: Dynamic Hip Screw.

A closed wedge osteotomy on the lateral side was then performed. The upper cut was made transversely, positioned 2 cm below the screw, while the lower cut was angled to complement the wedge configuration of the upper cut, converging on the inner surface of the femoral shaft. The resected bone piece was subsequently utilized as a graft at the osteotomy site. The distal fragment was displaced laterally and set in valgus, followed by fixation (Figure 4).

Closure of the wound was performed in layers after the insertion of a suction drain, which was removed after 48 hours. Skin staples were removed on the 14th postoperative day, and sterile dressing was applied.

Postoperatively, quadriceps and ankle pump exercises were initiated as soon as the patient was able to tolerate them. The patient was permitted to sit up, and mobilization was gradually started with partial weight-bearing. Full weight-bearing was permitted only after the osteotomy site showed radiological features of complete union.

Functional outcomes were assessed using the modified Harris Hip Score at four weeks, three months, six months, and twelve months postoperatively. Radiographic evaluations focused on union at the osteotomy site, femoral head viability, and any potential implant cut-out. Union was defined by the presence of trabeculations across the fracture or osteotomy site.

## Results

A total of 30 patients were included in the study, with 16 in the DABP group and 14 in the double-angle DHS group (Table 1). Both groups demonstrated improvement in HHS at 6-month and 12-month follow-up, but the double-angle DHS group showed a slightly higher HHS, indicating faster functional recovery. The angle blade plate showed better stability and required less bone removal. It allowed room for fibular grafting and early mobilization due to reliable fixation. However, it was more technically demanding and a less forgiving implant compared to DHS, which is simpler to use and provides compression at the fracture site. Despite this, the healing time was similar in both groups. Thus, the results were markedly satisfactory, with good functional outcomes, active painless hip range of motion, and painless weight-bearing with acceptable shortening in cases managed with either double-angle DHS or DABP, though each method had some shortcomings (Table 2). The results compared DHS and DABP based on key clinical parameters. Statistically significant differences were observed in post-op N-S angle (p = 0.042), pre-op Pauwels angle (p = 0.009), pre-op LLD (p = 0.003), post-op LLD (p = 0.048), pre-op HHS (p = 0.018), and post-op HHS (p = 0.002), favouring DABP in some aspects. Surgery time (p < 0.001), mobilization time (p < 0.001), and radiation exposure (p < 0.001) were significantly higher in the DABP group. No significant differences were found in blood loss (p = 1.000) or fracture union time (p = 0.229). Overall, DABP required a longer recovery period but maintained better post-op alignment, while DHS facilitated faster mobilization with less radiation exposure.

**Table 1:** Patient demographics. DHS: Dynamic Hip Screw; DABP: Double-Angled Blade Plate.

Serial No.	Age	Gender	Time of Presentation Since Injury (Weeks)	Treatment Method
1	51	Female	6	DABP
2	49	Male	6	DABP
3	31	Female	4	DHS
4	50	Female	5	DABP
5	46	Female	6	DABP
6	32	Male	6	DHS
7	38	Male	4	DHS
8	47	Male	6	DHS
9	54	Male	5	DABP
10	53	Female	5	DABP
11	49	Male	4	DABP
12	39	Female	5	DABP
13	39	Female	4	DABP
14	33	Male	5	DABP
15	35	Male	5	DABP
16	34	Male	5	DHS
17	56	Male	6	DABP
18	46	Male	4	DABP
19	26	Female	4	DHS
20	56	Male	4	DHS
21	27	Female	6	DHS
22	46	Male	4	DHS
23	58	Female	6	DABP
24	28	Male	4	DHS
25	52	Male	4	DHS
26	53	Female	6	DHS
27	29	Male	4	DHS
28	52	Male	4	DHS
29	43	Male	6	DABP
30	45	Female	5	DABP

**Table 2:** Comparison of postoperative results between the two groups. DADHS: Double-angled dynamic hip screw; DABP: Double-angled blade plate; HHS: Harris hip score; LLD: Limb length discrepancy. Values were compared using the means of postoperative results. t = t-test statistic (for independent samples t-test) p-value: Indicates statistical significance p < 0.05 (*): Statistically significant p < 0.01 (**): Highly significant p > 0.05: Not statistically significant

Parameter	DHS (Mean ± SD)	DABP (Mean ± SD)	Test Statistic	p-value
Pre-op N-S angle (°)	102 ± 5	105 ± 6	t = 1.98	0.054
Post-op N-S angle (°)	132 ± 4	135 ± 5	t = 2.15	0.042*
Pre-op Pauwels angle (°)	54 ± 3	50 ± 4	t = 2.72	0.009**
Post-op Pauwels angle (°)	30 ± 2	32 ± 3	t = 1.67	0.098
Pre-op LLD (cm)	2.5 ± 0.4	2.0 ± 0.3	t = 3.14	0.003**
Post-op LLD (cm)	0.5 ± 0.2	0.7 ± 0.2	t = 2.05	0.048*
Pre-op HHS (Harris hip score)	65 ± 6	70 ± 7	t = 2.41	0.018*
Post-op HHS (Harris hip score)	88 ± 5	82 ± 6	t = 3.29	0.002**
Mean blood loss (mL)	350 ± 30	350 ± 35	t = 0.00	1
Mean surgery time (minutes)	70 ± 10	90 ± 12	t = 5.01	<0.001**
Mean time to mobilization (hours)	48 ± 8	72 ± 10	t = 7.45	<0.001**
Mean time to fracture union (weeks)	13 ± 2	14 ± 3	t = 1.22	0.229
Mean time to osteotomy site union (weeks)	12 ± 2	13 ± 2	t = 1.52	0.136
Radiation exposure (mean no. of shots)	25 ± 5	45 ± 6	t = 10.56	<0.001**

**Figure 5:**
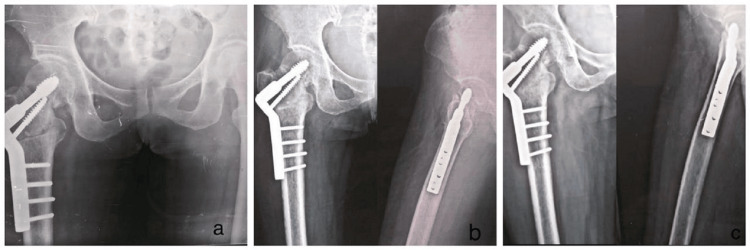
Follow-up radiographs for DHS patient. (a) Immediate postoperative X-ray; (b) Follow-up at 4 weeks; (c) Follow-up at 8 weeks. DHS: Dynamic Hip Screw.

**Figure 6:**
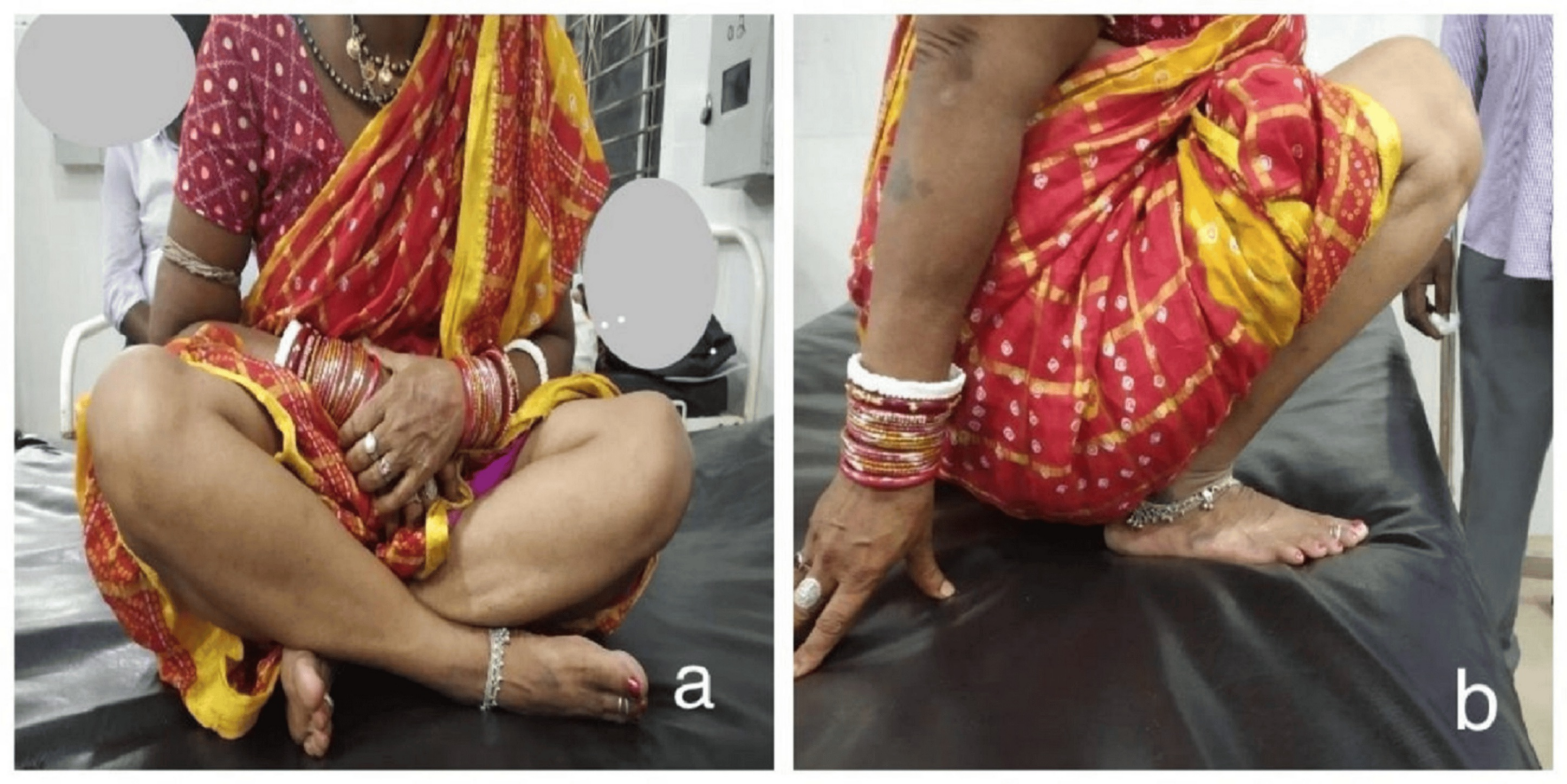
Clinical photographs of a patient treated with DHS. DHS: Dynamic Hip Screw.

**Figure 7:**
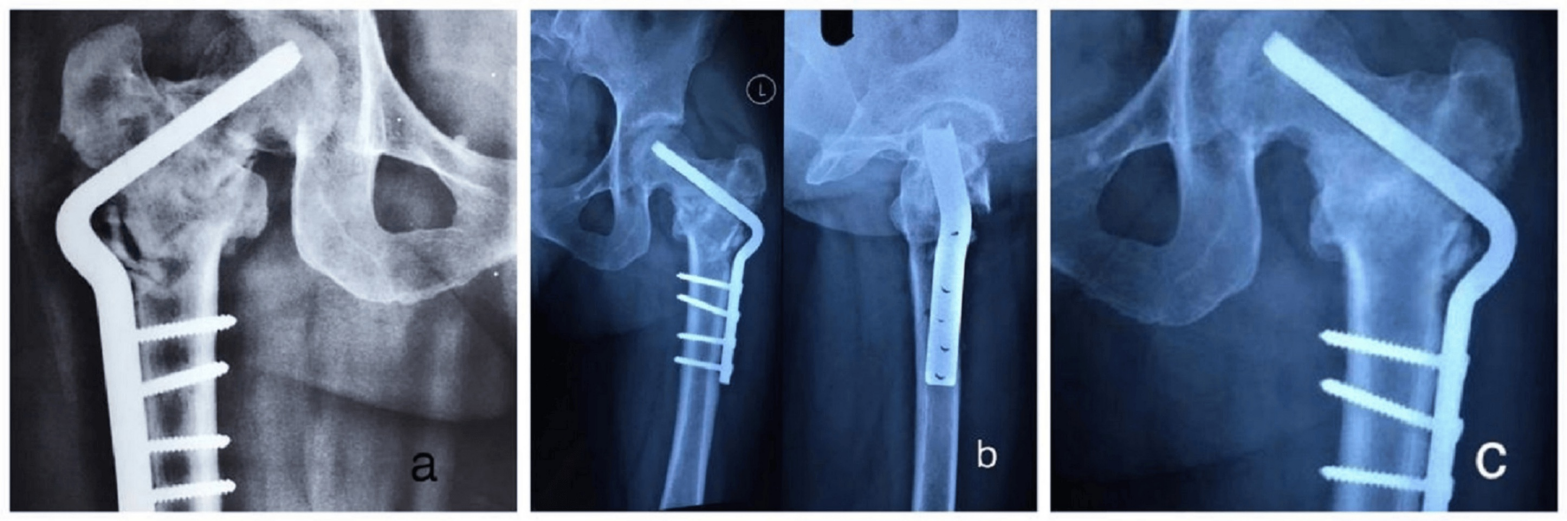
Follow-up radiographs for DABP patient. (a) Immediate postoperative X-ray; (b) Follow-up at 4 weeks; (c) Follow-up at 8 weeks. DABP: Double-Angled Blade Plate.

**Figure 8:**
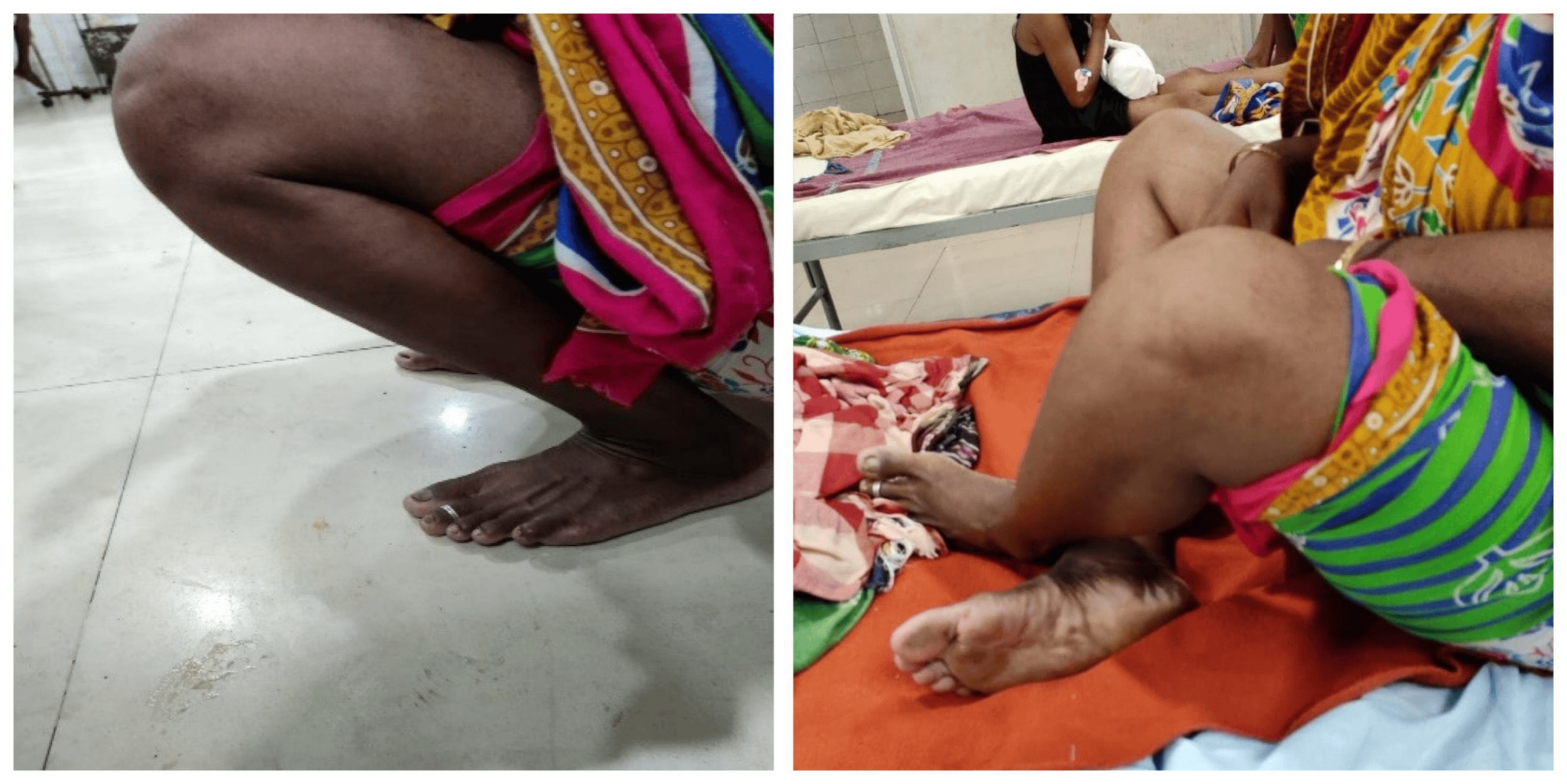
Clinical photographs of a patient treated with DABP. DABP: Double-Angled Blade Plate.

Our study demonstrated fixation with either double-angle DHS or DABP following Pauwels osteotomy for neglected neck of femur fractures. Adequate and appropriate physiotherapy was initiated from the second postoperative day, as tolerated by the patient. Patients were examined both radiologically and clinically at four weeks postoperatively, and every three weeks thereafter until union and full weight-bearing were achieved (Figures 5-8). 

**Figure 9:**
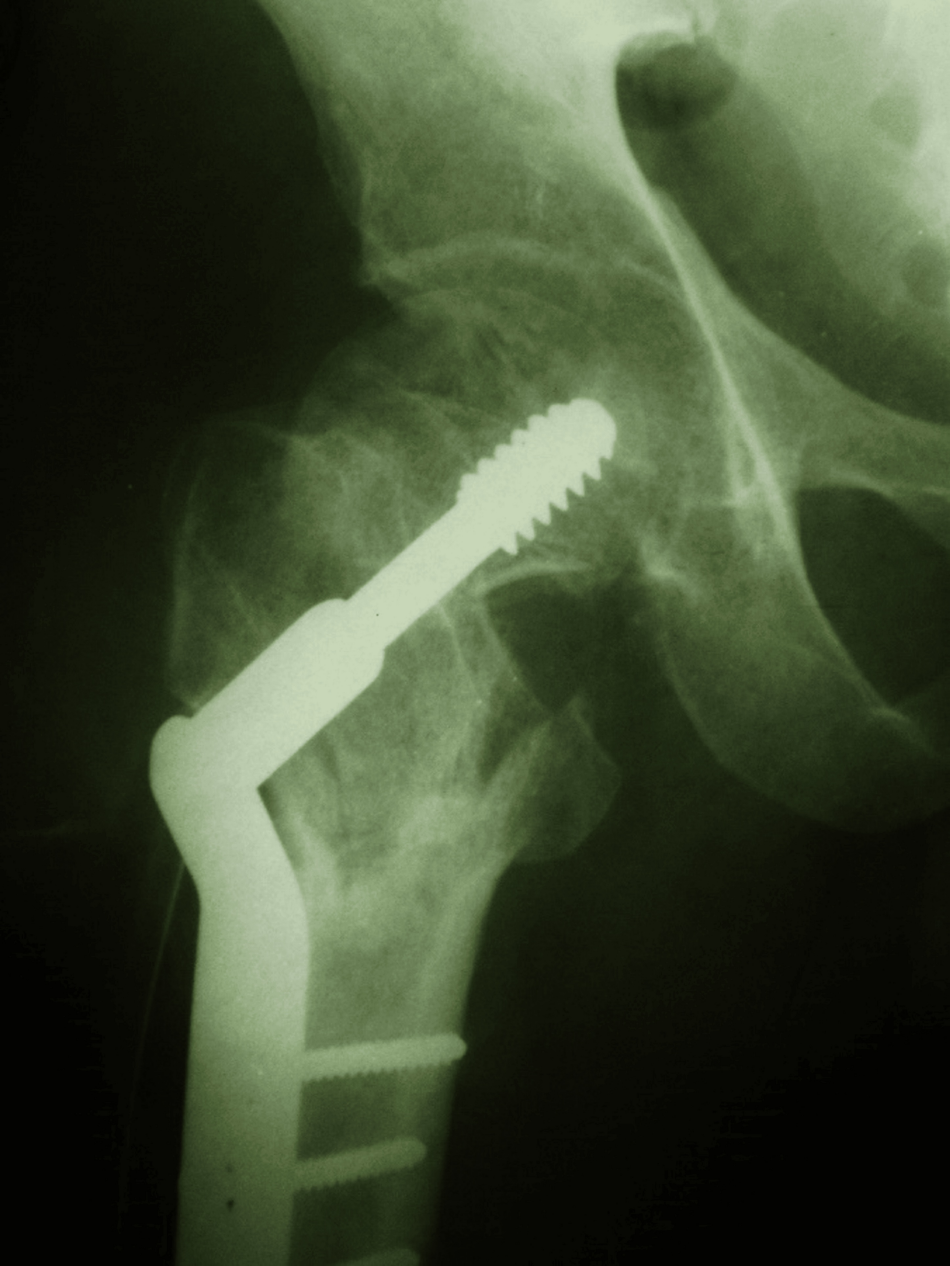
Osteonecrosis in a patient treated with DHS. DHS: Dynamic Hip Screw.

**Figure 10:**
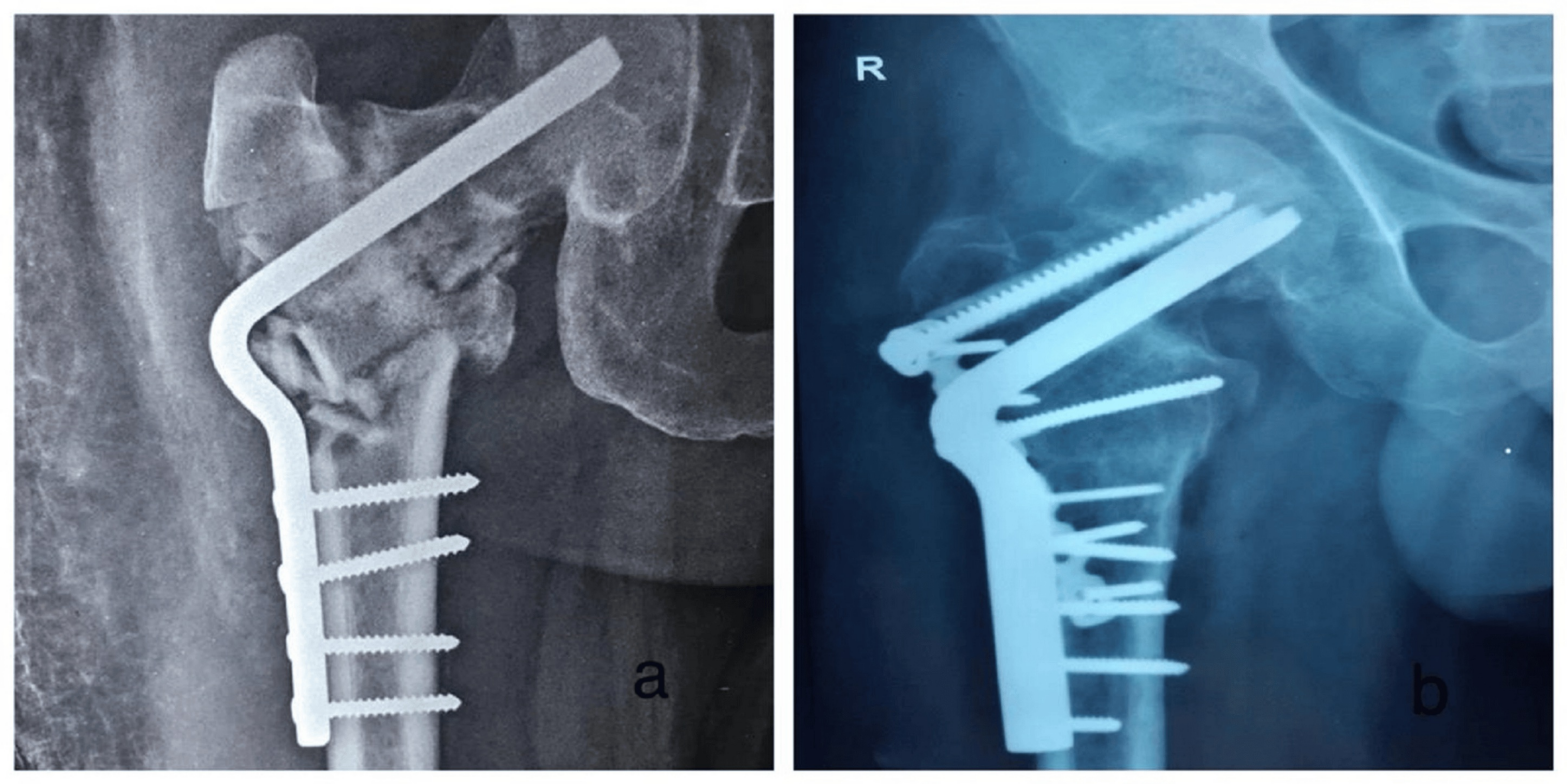
Fracture in a DABP case managed with revision surgery. DABP: Double-Angled Blade Plate.

Few complications were observed in the study. Notably, one patient in the DHS group developed osteonecrosis of the femoral head (Figure 9). Additionally, in the DABP group, another patient experienced an iatrogenic fracture, which was subsequently managed through revision surgery (Figure 10).

## Discussion

Fractures of the neck of the femur are among the most serious injuries affecting the elderly population [1], often resulting from falls or underlying bone fragility. Given their complex anatomy and the high risk of complications such as avascular necrosis and non-union, these fractures present significant challenges to orthopaedic surgeons. The term neglected is used to indicate a delay of 30 days or more, according to Meyers MH et al. [3]. In the management of neglected femoral neck fractures in young adults, achieving fracture union and restoring functional mobility are paramount. This discussion compares the outcomes of internal fixation using a DABP versus a double-angle DHS combined with Pauwels osteotomy, evaluating their effectiveness in terms of fracture union, limb length discrepancy, and functional outcomes.

Internal fixation with DABP and Pauwels osteotomy

The DABP has long been employed for stabilizing femoral neck fractures due to its robust fixation capability. Its design facilitates rigid fixation and maintains alignment by bridging the fracture site. It also provides space for fibular grafting to aid in union. Despite its advantages, studies have highlighted several challenges. The primary issue with the blade plate is its complexity in achieving precise alignment and adequate compression. Additionally, there is a risk of complications such as screw cut-out and non-union, especially in high-angle Pauwels fractures where the mechanical stress on the plate can be considerable [8,9].

Internal fixation with double-angle DHS and Pauwels osteotomy

The double-angle DHS combined with Pauwels osteotomy represents a refined approach for managing these fractures. Pauwels osteotomy, a technique designed to alter the mechanical load on the femoral neck, aims to improve the healing potential by correcting the Pauwels angle to less than 30°, thus enhancing the chances of union [10]. This technique adjusts the alignment of the fracture site and optimizes load distribution, which is particularly beneficial in cases of high Pauwels angles where traditional fixation methods might fail.

A key finding of our study was that the combination of Pauwels osteotomy with double-angle DHS yielded a higher success rate in achieving fracture union compared to internal fixation with the DABP. This is consistent with previous research indicating that modifying the Pauwels angle can significantly improve the biomechanical environment at the fracture site, facilitating better healing [11,12]. The DHS system’s design, which includes lag screws and a side plate, offers a stable fixation that supports compression at the fracture site, thereby promoting union.

Limb length discrepancy is a common concern in the surgical management of femoral neck fractures. The study demonstrated that both methods resulted in acceptable limb length discrepancies; however, the double-angle DHS combined with Pauwels osteotomy often provided more precise control over limb alignment. The ability to perform a controlled osteotomy and accurately align the limb reduces the risk of significant length discrepancies, a critical factor in achieving satisfactory functional outcomes [13].

Functional recovery was another area where the double-angle DHS with Pauwels osteotomy showed marginally superior results. Functional outcomes were assessed using the modified HHS, which revealed better scores in patients treated with the DHS system. This can be attributed to the enhanced stability provided by the DHS and the improved alignment of the femoral head achieved through Pauwels osteotomy. Studies have consistently shown that better alignment and stable fixation lead to improved range of motion and reduced pain, translating into higher functional scores [14,15].

Complications are an important aspect to consider when comparing these techniques. While both methods had complications, including a case of osteonecrosis in the DHS group and an iatrogenic fracture in the blade plate group, the overall complication rate was relatively lower with the double-angle DHS and Pauwels osteotomy. The latter approach’s ability to address high Pauwels angles more effectively may contribute to a reduced incidence of complications related to non-union and implant failures [16].

Limitations

The study’s limitations include its small sample size of only 30 patients, which may not provide sufficient statistical power to make broad generalizations. The absence of long-term follow-up also limits the ability to assess the durability and long-term outcomes of the two fixation methods. Furthermore, variations in surgeon experience and technique might have influenced the results, restricting the study’s ability to provide a definitive comparison. Finally, the study’s focus on a single institution may affect the generalizability of its findings to other clinical settings. Further multi-centric studies are recommended to validate the present outcomes.

Conclusion

In conclusion, this study demonstrates that internal fixation utilizing double-angle DHS in conjunction with Pauwels osteotomy results in a significantly higher success rate in terms of fracture union. Furthermore, this approach maintains acceptable limb length discrepancies and yields enhanced functional outcomes in young adults with neglected femoral neck fractures. The method represents a valuable intervention that offers superior results for appropriately selected patients.

The results of our study are consistent with existing literature advocating the combination of osteotomy and contemporary fixation techniques to optimize fracture management. Accordingly, for young adults presenting with neglected femoral neck fractures, the application of double-angle DHS alongside Pauwels osteotomy should be regarded as a safe and preferred strategy to improve clinical outcomes.

## Conclusions

This study concluded that Pauwels osteotomy combined with fixation using a double-angle DHS provided a relatively higher success rate in achieving union, with acceptable limb length discrepancy, better functional outcomes, and easy implant availability. This makes it a valuable head-salvaging procedure for young adults with neglected femoral neck fractures when appropriately selected.
